# Comentário sobre o artigo “Maria José Deane por ela mesma e por nós”^*^

**DOI:** 10.1590/S0104-59702026000100029

**Published:** 2026-08-10

**Authors:** Francisco José Roma Paumgartten

**Affiliations:** i Departamento de Ciências Biológicas/Escola Nacional de Saúde Pública/Fiocruz. Rio de Janeiro – RJ – Brasil. francisco.paumgartten@gmail.com

A recente publicação da entrevista concedida por Maria José von Paumgartten Deane em 1989 é oportuna (cf. Maciel, Borges, 2025). Trata-se de documento em que a cientista fala do preconceito de gênero, o “mito da superioridade masculina”, do questionamento dos dogmas da religião católica, dos desafios da maternidade e questões íntimas, como a manutenção da virgindade antes do casamento. A entrevistada discorre sobre a infância, ancestralidade paterna e materna, primeiros estudos no Colégio Paumgartten, secundário no Ginásio Paes de Carvalho e superior na Faculdade de Medicina e Cirurgia do Pará. Em 1937, a acadêmica de medicina aderiu voluntariamente ao grupo de jovens pesquisadores do Instituto de Patologia Experimental do Norte e do Instituto Oswaldo Cruz que, sob a liderança de Evandro Chagas, se aventurou nas selvas de Abaeté em busca de casos autóctones de calazar. A linha de tempo da narrativa é interrompida bruscamente em 1975, quando se abordou a prisão da filha na Argentina. O áudio registra as palavras finais da entrevistada: “A capacidade que tem o indivíduo humano de ser cruel, é incrível, incrível. Olha, já acabou, agora vocês vão embora, me despeço, contei minha história” (Deane, 2024, p.25). Revolver, 14 anos depois, as recordações daqueles dolorosos eventos fizeram-na encerrar a entrevista.

**Figura 1:**
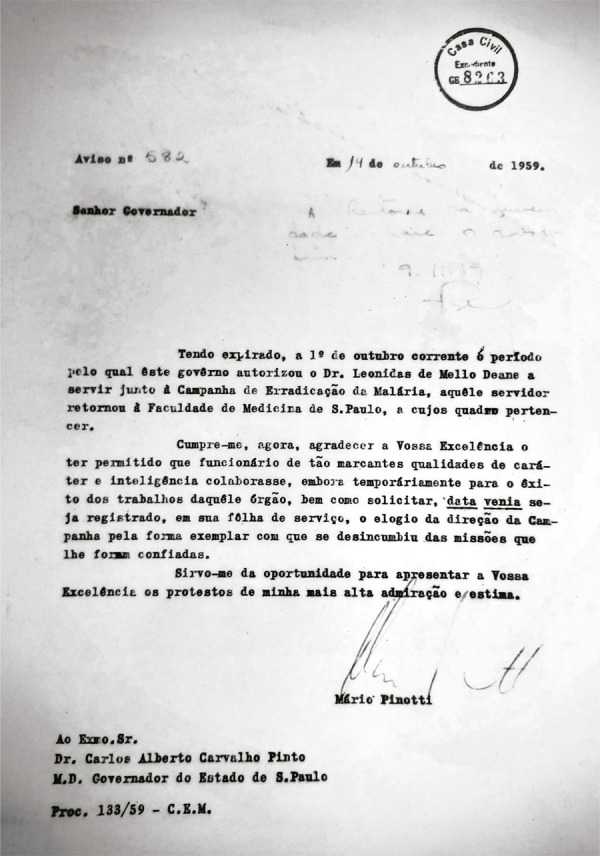
: Ofício de Mário Pinotti solicitando ao governador de São Paulo que registrasse elogio na folha de serviço de Leônidas de Mello Deane, 14 de outubro de 1959 (Fonte: fotocópia em arquivo de Francisco J.R. Paumgartten)

Alguns erros e imprecisões empanam o brilho dessa contribuição à memória de Maria Deane. Ela não “ingressou na Faculdade de Medicina e Cirurgia do Pará em 1936”, mas em 1932, e colou grau em 1937. Maria Deane contribuiu para o estudo pioneiro da filariose bancroftiana em Belém e evidenciou, pela primeira vez no Brasil, infecções pela *Mansonella ozzardi* em Manaus, mas não estudou filarioses em outros estados (Paumgartten, Oliveira, 2025). A transmissão da malária na região da Estrada de Ferro Vitória-Minas (EFVM), no Espírito Santo e em Minas Gerais, foi alvo de uma inspeção do casal Leônidas e Maria Deane, que avaliou, em janeiro e fevereiro de 1944, o progresso das ações de controle da endemia no vale do rio Doce, compromisso que o Serviço Especial de Saúde Pública assumiu em 1943. Pela EFVM escoavam minerais estratégicos para o esforço de guerra americano. A assertiva “foi chefe do laboratório de entomologia da Campanha de Erradicação da Malária, do Ministério da Saúde, até 1961 quando ingressou no Instituto de Medicina Tropical, da Universidade de São Paulo (USP)” (Maciel, Borges, 2025, p.3) repete o que é difundido em notas biográficas sobre Maria Deane, mas não corresponde à realidade. Cedidos pela Universidade de São Paulo (USP) à Campanha de Erradicação da Malária (CEM), sediada na então capital federal, os Deane residiram no Rio de Janeiro de outubro de 1958 a setembro de 1959. Mário Pinotti, antigo conhecido dos Deane e, entre 1958 e 1960, ministro da saúde, incumbiu o casal de organizar o Laboratório Central da CEM e estabelecer planos de treinamento do pessoal do serviço para a rotina e pesquisa. Nesse período, Leônidas ausentou-se com frequência do Rio, inclusive em abril de 1959, para participar do Curso de Técnicas Entomológicas Avançadas na Escola de Higiene e Medicina Tropical da Universidade de Londres, onde aprendeu o método de determinação da idade fisiológica de mosquitos desenvolvido pela pesquisadora russa Tatiana Sergeevna Detinova. Ao retornar, em junho de 1959, treinou entomologistas da CEM a empregar esse método de grande utilidade para a campanha antivetorial em curso. Em virtude das outras ocupações do marido, recaiu quase inteiramente sobre Maria Deane a responsabilidade de implantar o laboratório de entomologia. Em 14 de outubro de 1959, Pinotti encaminhou ao governador de São Paulo, Carlos Alberto Carvalho Pinto (1910-1987), ofício agradecendo os serviços prestados por Leônidas e solicitando que fosse registrado na sua folha de serviço um elogio da direção da Campanha (Figuras 1 e 2). O ano no Rio foi agradável para a família. Exceto os Deane que ainda moravam em Belém, os demais familiares de Maria e Leônidas residiam na cidade. As minhas recordações de infância registram uma visita ao laboratório de entomologia. Lembro-me dos insetários e das explicações da minha tia sobre como alimentava mosquitos e barbeiros. Mostrou-me larvas e ovos de mosquitos ao microscópio, e saciou a minha curiosidade sobre os barbeiros, levando-me, aos 8 anos, a entender o que era xenodiagnóstico.

**Figura 2:**
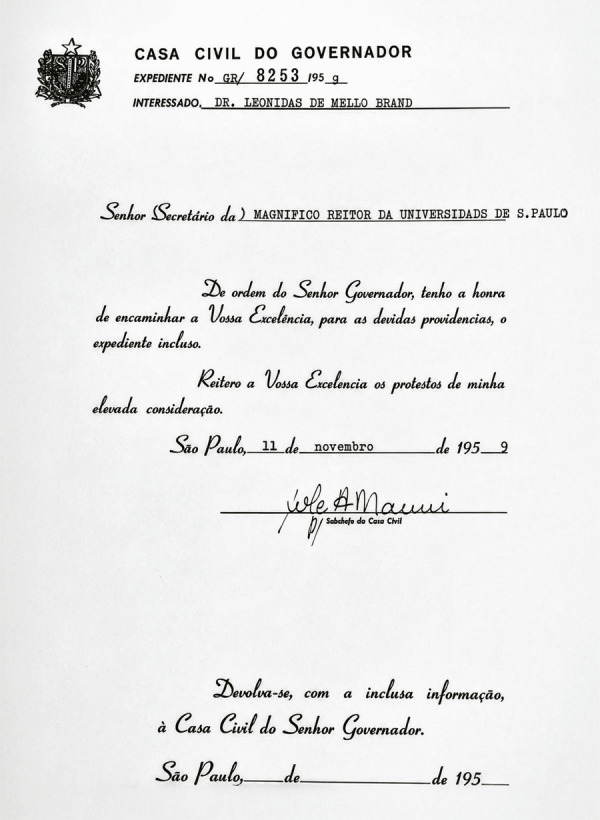
: Encaminhamento ao reitor da USP do ofício do ministro Pinotti, 11 de novembro de 1959 (Fonte: fotocópia em arquivo de Francisco J.R. Paumgartten)

O Instituto de Medicina Tropical de São Paulo (IMTSP), inicialmente vinculado à Faculdade de Medicina da USP, e o respectivo periódico foram criados por Jânio Quadros, em 15 de janeiro de 1959 (decreto 34.510), época em que o casal residia no Rio. Samuel Pessoa, aposentado desde 1956, foi um dos mais firmes apoiadores da iniciativa de Carlos Lacaz, e vários de seus antigos assistentes no Departamento de Parasitologia prontamente migraram para o IMTSP, incluindo Maria Deane e Luis Rey, que iria se tornar o primeiro editor da revista do IMTSP. Entre outubro de 1959 e 1961, Leônidas assumiu interinamente a chefia do Departamento de Parasitologia da Faculdade de Medicina da USP. Antônio Dácio Franco do Amaral, o sucessor de Pessoa, fora contratado para criar a cátedra de Parasitologia na Universidade de Carabobo na Venezuela, e licenciara-se. Em outubro de 1959, Maria Deane transferiu-se para o IMTSP, onde, nos anos 1960, estudou a biologia de tripanossomatídeos.

Reparos são necessários também em relação às afirmações que as autoras atribuem à palestra que, em novembro de 1995, Luis Hildebrando proferiu em memória de Maria Deane: “Os últimos 15 anos de vida foram os mais profícuos de sua trajetória profissional, após a transferência para o IOC em 1980” (Maciel, Borges, 2025, p.4). Hildebrando afirmou: *“*Os últimos 10 a 15 anos da vida profissional de Maria Deane foram, creio eu, os momentos mais reconfortantes e agradáveis de sua trajetória como pesquisadora ... graças ao ambiente sério, porém cordial, amoroso, confiável e de apoio criado ao seu redor no Instituto Oswaldo Cruz, primeiro por José Rodrigues Coura e depois por Carlos Médicis Morel, ‘os Deanes’ puderam retomar suas atividades” [“The last 10 to 15 years of Maria Deane’s professional life were, I suppose, the most comforting and agreable moments of her life as a researcher ... thanks to the serious but cordial, loving, reliable, supportive atmosphere created around them at the Oswaldo Cruz Institute, first by José Rodrigues Coura, and later by Carlos Médicis Morel, ‘the Deanes’ could begin again their activity”] (da-Silva, 1996, p.2). Como se vê, Hildebrando especulou que os últimos 15 anos de vida teriam sido os mais agradáveis da carreira da pesquisadora, mas não se referiu à sua produção científica. A contribuição dos Deane à parasitologia e Medicina Tropical deu-se, essencialmente, em três instituições brasileiras: o Instituto Evandro Chagas, a USP e o Instituto Oswaldo Cruz. Hildebrando conhecia bem os trabalhos de Maria Deane na USP com Judith Kardos Kloetzel (1927-2015), uma das suas melhores amigas, e Regina Vugman Milder, e não ousou julgar qual período da trajetória teria sido “o mais profícuo”. A comparação não fazia sentido.

Em 1976, José Witremundo Torrealba Tovar (1935-1981) convidou os Deane a desenvolver pesquisas no Departamento de Parasitologia da Facultad de Ciencias de la Salud da Universidad de Carabobo. Torrealba, ex-aluno da Faculdade de Medicina da USP, graduou-se em 1959, e tornou-se decano da faculdade venezuelana. Maria Deane respondeu que o casal aceitaria o convite se ele conseguisse documentos para a filha (Paumgartten, Oliveira, 2025). Após algum tempo, eles receberam em Lisboa correspondência com um passaporte em branco e instruções para se dirigir ao consulado da Venezuela, onde o documento foi preenchido com os dados de Luisa. O governo de centro-esquerda de Carlos Andrés Pérez (1922-2010) anuiu com o convite, sem o qual Torrealba não conseguiria o passaporte. Partiu, porém, de Witremundo Torrealba a iniciativa de convidar o casal. Ele e o pai, José Francisco Torrealba (1896-1973), foram médicos tropicalistas de enorme prestígio no meio acadêmico da Venezuela.

No preâmbulo da entrevista, as autoras afirmaram que “ambos não autorizaram seu acesso público depois de finalizadas”, empecilho que teria sido contornado 35 anos depois (Maciel, Borges, 2025, p.3). Não obstante, em 1994, a *História, Ciências, Saúde – Manguinhos* publicou artigo reproduzindo trechos da entrevista de Leônidas (Britto et al., 1994). O casal nunca impôs restrições de acesso às entrevistas. Concederam-nas porque desejavam tornar pública a história de vida e o que pensavam. Consentiram tacitamente, quando não era praxe assinar documento formal de autorização. Ao aplicar retroativamente e equivocadamente a lei de proteção de dados pessoais de 2018, não se protegeu, mas frustrou as expectativas dos entrevistados.

Em editorial em tributo à Maria Deane, Hildebrando, talentoso escritor, reconstruiu um diálogo ficcional que capta, espirituosamente, uma das questões que obcecou a cientista até os últimos dias. Como teria evoluído a relação entre parasitos e seus hospedeiros? O diálogo envolve o *Polychromophilus deanei* descoberto pelo casal em morcegos da Amazônia e o que isso significaria em termos do desembarque da malária no Novo Mundo. A outra foi a constatação do duplo ciclo do *Trypanosoma cruzi* no gambá em 1984. O achado não esclarece a “dinâmica da transmissão oral”, e a sua importância vai além de explicar a ocasional transmissão da doença de Chagas sem a participação de barbeiros (Maciel, Borges, 2025, p.3). Ele subsidia a elegante hipótese da glândula anal do marsupial ter sido o *locus* evolucionário onde um ancestral do *cruzi* evoluiu de parasito monogenético de insetos para um digenético cujo ciclo de vida se dá em dois hospedeiros: barbeiros e vertebrados.

Não poderia nesta carta me estender sobre todos os erros e imprecisões difundidos nas notas biográficas sobre os Deane, mas corrigi-las, na medida do possível, faz jus à memória deles.
